# A dayside aurora dataset from the Global-scale Observations of the Limb and Disk mission

**DOI:** 10.1038/s41597-026-06884-2

**Published:** 2026-02-19

**Authors:** Jordan Holmes, Scott L. England

**Affiliations:** Aerospace and Ocean Engineering, Virginia Polytechnic Institute and State University, Blacksburg, Virginia USA

## Abstract

We present a comprehensive dataset of dayside auroral emissions observed by the Global-scale Observations of the Limb and Disk (GOLD) mission from October 2018 to June 2025. The dataset contains over 47,000 unique scans of the northern aurora in three far-ultraviolet spectral channels (OI 135.6 nm, NI 149.3 nm, and N₂ LBH), estimates of the background dayglow, binary masks of auroral locations, and other corresponding spatial and temporal metadata. The OI 135.6 nm, NI 149.3 nm, and N₂ LBH emissions are far-ultraviolet signatures of electron-impact excitation in the upper atmosphere and therefore serve as tracers of auroral electron precipitation. From this dataset, auroral pixels are directly available with no dayglow contamination of the emissions. Auroral signals are extracted through a multi-stage processing pipeline inspired by computer vision and machine learning techniques. This dataset provides a consistent view of the dayside aurora over the North American and Atlantic sectors, enabling studies of auroral dynamics with GOLD observations.

## Background & Summary

The aurora is one of the most visually striking manifestations of space weather; driven by the precipitation of charged particles from various regions of the Earth’s magnetosphere and solar wind into the upper atmosphere. As these particles enter the ionosphere–thermosphere (IT) system, they deposit energy that alters neutral and plasma dynamics^1,2^. The aurora’s occurrence provides an indicator of magnetospheric coupling with the IT system. Changes in the location, brightness, and spectrum of the aurora are linked to geospace disturbances that can cause significant societal impacts including ground-based power outages, disruptions to radio wave propagation, and anomalies in spacecraft operation^3^.

Although visible auroral displays are often appreciated as nighttime phenomena, auroras occur continuously throughout the day and night. Dayside auroral observations are particularly valuable for understanding solar wind-magnetospheric interactions and monitoring the dynamics of magnetic reconnection at the magnetopause^4^. However, dayside auroral observations have historically presented challenges due to the overwhelming solar induced emissions (dayglow) that can mask or obscure auroral emissions.

Space-based far-ultraviolet (FUV) imaging has emerged as the primary technique for dayside auroral observations, as FUV emissions can be distinguished from background dayglow, and are not overpowered by reflected light from the surface of the Earth (which impacts visible wavelengths). Over the past two decades, instruments including the spectrographic imager (SI) and widefield imaging camera (WIC) onboard IMAGE^5,6^, the Global Ultraviolet Imager (GUVI) onboard TIMED^7,8^ and the Special Sensor Ultraviolet Spectrographic Imager (SSUSI) on multiple DMSP satellites (2003-present) have provided valuable UV auroral datasets^9^. These measurements have excelled at providing global-scale coverage and high spatial resolution observations, offering crucial insights into auroral precipitation patterns.

**Fig. 1:**
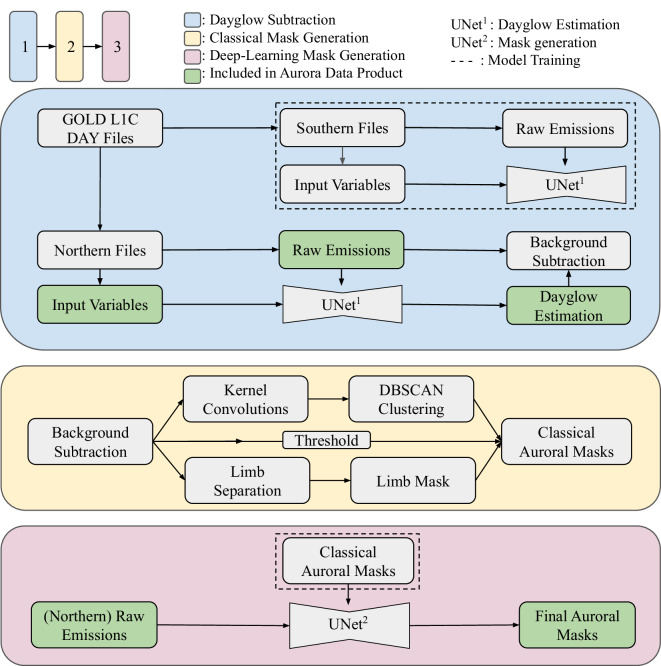
Schematic overview for generating the dayside auroral dataset. Step 1 (blue) shows the steps taken to train a UNet^20^ model and to remove the dayglow signal from the raw northern scans. Step 2 (yellow) applies signal processing techniques to produce binary masks of the aurora. Step 3 (pink) uses the classical masks to train a secondary UNet model to produce the final dayside auroral pixel locations. Green boxes indicate data that is kept in the final product.

The Global-scale Observations of the Limb and Disk (GOLD) mission provides a complementary observational capability that builds upon these datasets through a fundamentally different perspective. Launched in 2018 and positioned at 47.5°W geostationary orbit, GOLD provides continuous monitoring of Earth’s disk from a fixed vantage point in the Far UV (FUV) range^10^. This geostationary perspective enables repeated imaging of the same geographic regions, making it uniquely suited for tracking auroral temporal evolution and dynamics. In 2020, Michell^11^ demonstrated the instrument’s capability to observe the aurora in two case studies. Inspired by this, but realizing the challenge in applying the prior methodology to the broader dataset, we developed our own scaled and automated approach to collect available dayside auroral observations from October 2018 - June 2025, shown schematically in Fig. 1.

## Dataset Description

This comprehensive auroral image dataset is derived from the GOLD Level 1 C (L1C) “DAY” products spanning October 2018 to June 2025. The dataset contains over 47,000 unique observations of the Northern hemisphere. Each observation in the dayside aurora dataset includes:

1. Raw hemispheric scans of three spectral bands covering: 135.6 nm, 149.3 nm, and LBH which are key auroral emissions for OI, NI, and N_2_ respectively.
2. Dayglow background estimates: representing non-auroral contamination.
3. Binary masks identifying auroral pixel locations.
4. Spatial and temporal metadata (e.g. geographic latitude, magnetic local time).

Oxygen and molecular nitrogen dominate the neutral thermosphere, producing bright FUV emissions in the auroral region. The NI 149.3 nm emission is also bright and closely tracks the spatial structure of the N_2_ LBH emission, as the NI emission arises from electron impact dissociation of N_2_^12^. Together, these three wavelengths serve as markers of enhanced auroral electron precipitation zones.

## Scientific Applications and Reuse Value

This dataset provides large-scale information of the dayside aurora with significant reuse potential for the space weather community. This dataset removes the data processing barrier necessary for conducting dayside auroral research, as seen from GOLD. Specific potential applications include: (1) statistical analysis of auroral boundary information and its relationship to solar wind and geomagnetic parameters, (2) validation of magnetosphere-ionosphere coupling models, (3) development of a deep learning auroral forecasting model with data from multiple missions. This dataset extends the utility of GOLD by providing accessible auroral observations that can be readily used in conjunction with other datasets for comparative or multimodal studies.

## Methods

To construct a high-quality dataset of dayside auroral emissions from GOLD observations the next sub-sections discuss:

1. Observations from the GOLD mission.
2. Removal of dayglow contamination by leveraging southern hemispheric observations.
3. Application of classical computer vision, clustering, and signal processing techniques to segment auroral regions, producing an intermediate, automatically-labelled dataset.
4. Utilization of this intermediate dataset (3) to train a deep learning model (UNet), improving segmentation across storm conditions and during periods of reduced GOLD cadence in later years.

Below we describe each stage in detail.

### GOLD raw observations & characteristics

The dayside aurora dataset is derived from the GOLD Level 1 C DAY (L1C DAY) products^13,14^. Each file corresponds to a single hemispheric scan with row-column dimensions of 52 × 92 non-zero pixels and a spectral dimension of 800 channels, spanning 132–162 nm. Each file (hemispheric scan) is produced by sweeping the entrance slit over an entire hemisphere, gathering spectral information at each pixel; this process takes approximately 12 minutes from start to end, with each pixel considered as a snapshot. Integrating across the spectral dimension can produce an image of a hemisphere, and three auroral emissions can be resolved (Table 1).

**Table 1:** Observable auroral species and the GOLD L1C DAY radiance band definitions^25^.

Feature Name	Wavelength integration intervals [nm]
1356 (OI)	[135.0, 137.0]
1493 (NI)	[149.0, 149.8]
LBH (N_2_)	[137.7, 140.1], [140.9, 142.2], [142.5, 143.7], [144.2, 145.4], [146.1, 148.0], [149.9, 152.0], [152.8, 154.0]

Full disk images of the Earth can be created by combining paired northern and southern hemispheric scans, with a 12 minute difference between the start of the two hemispheric scans. Each full disk image covers geographic latitudes [−74°,74°] and longitudes[−127°, 32°]. Poleward of 60° latitude, the average spatial resolution is 264 km × 264 km with the largest being 345 km × 345 km. From 2018–2021, GOLD produced 34 full disk scans per day with a 30 minute cadence between subsequent full disk scans. After 2021, the cadence between subsequent full disk images decreased to 2 hours to extend the instrument’s lifetime due to sensor degradation^15^.

Due to GOLD’s geostationary orbit and longitude (47.5°W), and the location of the Earth’s geomagnetic poles, southern auroral emissions are rarely observed, except during major geomagnetic storms^16^. Southern scans at low geomagnetic activity therefore provide an aurora-free reference, dominated entirely by dayglow emissions. This apparent limitation is in fact a major strength: southern scans can be exploited to model and remove dayglow that is present in the northern scans, leaving just the aurora. Later, we address how periods of high geomagnetic activity are handled when aurora australis shifts to lower latitudes.

### The dayglow issue

The primary challenge in isolating dayside aurora is strong contamination from dayglow emissions, which are largely solar-driven radiances produced by photoelectron excitation of neutral species in the thermosphere^17^. The observed dayglow intensity largely depends on geometric and periodic quantities such as: solar zenith angle (SZA), emission angle (EMA), day of year (DOY), and to some extent geomagnetic activity (e.g., Kp^18^), all of which affect excitation, extinction, and line-of-sight integration.

In principle, the characterization of the dayglow can be done using physics-based models (e.g., GLOW^19^), but these approaches require precise instrument calibration and detailed atmospheric specification, which may limit their accuracy when applied to experimental data from GOLD. Instead, we adopt a data-driven approach so that the dayglow estimations are relative to the instrument’s own observations. Thus, southern scans are used as an empirical dayglow template for raw observations seen in northern scans. To account for Earth’s axial tilt relative to the ecliptic plane, southern scans are paired with northern scans 183 days apart, to account for seasonal changes in composition. We note that variations in solar flux can introduce additional interhemispheric differences; a method to correct for these differential solar conditions is proposed later. Near equinox, this requirement isn’t necessary. However, simple subtraction is still limited by the timing differences (~12 min) of the northern and southern scans that lead to systematic lighting differences. To overcome the timing issue, and to ensure the subtracted dayglow corresponds to the geomagnetic activity of the northern scan, we trained a deep learning model to predict dayglow directly from geometric and periodic inputs (SZA, EMA, DOY) and Kp.

The training data includes all southern hemispheric scans from 2020 to learn the geometric and periodic dependence of the dayglow since it is a year with a high cadence of measurements. 2020 is a geomagnetically quiet year, so these samples simultaneously learn the low geomagnetic dependence of the dayglow. To capture the moderate to high geomagnetic dependence of the observed dayglow, all southern scans with an associated Kp between 4 to 6 activity from 2019–2022 were also included in the model training set. Higher Kp intervals (>6) were excluded from the training set to avoid aurora australis contamination in the dayglow model. Consequently, model outputs during inference for high-Kp events are extrapolated beyond the training data and are less reliable.

The L1C DAY products contain the variables necessary to train a dayglow model. SZA and EMA values come in array format, matching the same spatial dimensions of an emission scan. DOY and Kp values are scalar values, so we expand these values to match the same spatial dimensions as SZA and EMA. Geometric and periodic variables were encoded using sine-cosine transformations (e.g., SZA → sin(SZA), cos(SZA)) instead of standard normalization. The nearest and preceding Kp values, retrieved from the PySpaceWeather package^18^, were used for model inputs to account for any possible time history effects; this feature was standardized to a mean of zero and standard deviation of one. Preprocessing for the outputs (southern emission scans) was a per-channel standardization across the entire dataset; no normalization was applied.

All missing values on the off disk and limb pixels, in the inputs and labels, were imputed with zeroes.

The deep learning model used to estimate the dayglow emission is a UNet, a type of convolutional neural network (CNN)^20^. UNets are particularly well suited for image-to-image problems, where the input and output share the same spatial structure. Since we have image-like inputs that match the same dimensions as the output, we employ this deep learning architecture to learn the dayglow relationship. This model was chosen for its applicability for different tasks in regression (for dayglow estimation), and segmentation (final part of methods). The UNet architecture largely follows the off the shelf architecture with additional padding to accommodate the rectangular spatial dimensions of the image. The model was trained with the AdamW optimizer (learning rate 1e^−3^, weight decay 1e^−2^), using mean squared error as the loss and early stopping (10 epochs) based on the validation R². Non-Earth pixels were masked, as these pixels aren’t important when considering dayglow emissions. The entire dataset was randomly split into training, validation, and test sets in a 60/20/20 ratio. The dayglow model explains 95% and 93% of the variance (R^2^ = 0.95 & R^2^ = 0.93) in the validation and test sets respectively, indicating a robust representation of the southern hemispheric data.

For inference, the northern hemispheric scan variables are used to estimate the dayglow. A scaling factor is determined via least-squares regression on low-latitude pixels to get the correct hemispheric-averaged intensity level on a given day; which accounts for small fluctuations in the solar extreme UV flux between the observed day and the corresponding data from the southern hemisphere used for dayglow subtraction. After scaling, the southern dayglow estimate is subtracted from the northern scan channel-wise, and only positive residuals are retained in the resulting differenced image.

### Classical segmentation

**Fig. 2:**
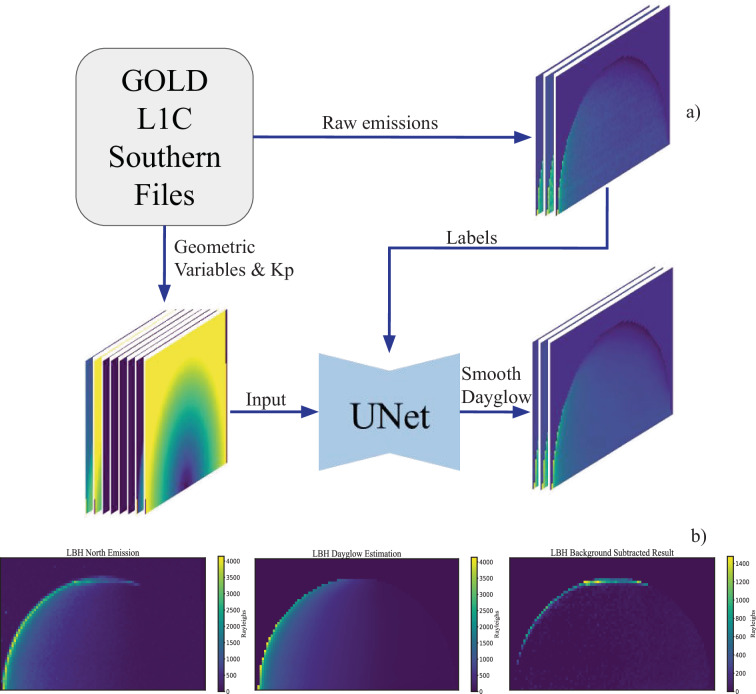
(**a**) Dataflow for training the dayglow UNet model. The standardized input variables are paired with raw southern hemisphere emissions. The UNet outputs smoothed representations of the dayglow. (**b**) Instance of the dayglow being removed from a northern scan. While some positive residuals remain near the limb, most of the dayglow is effectively removed.

**Fig. 3:**
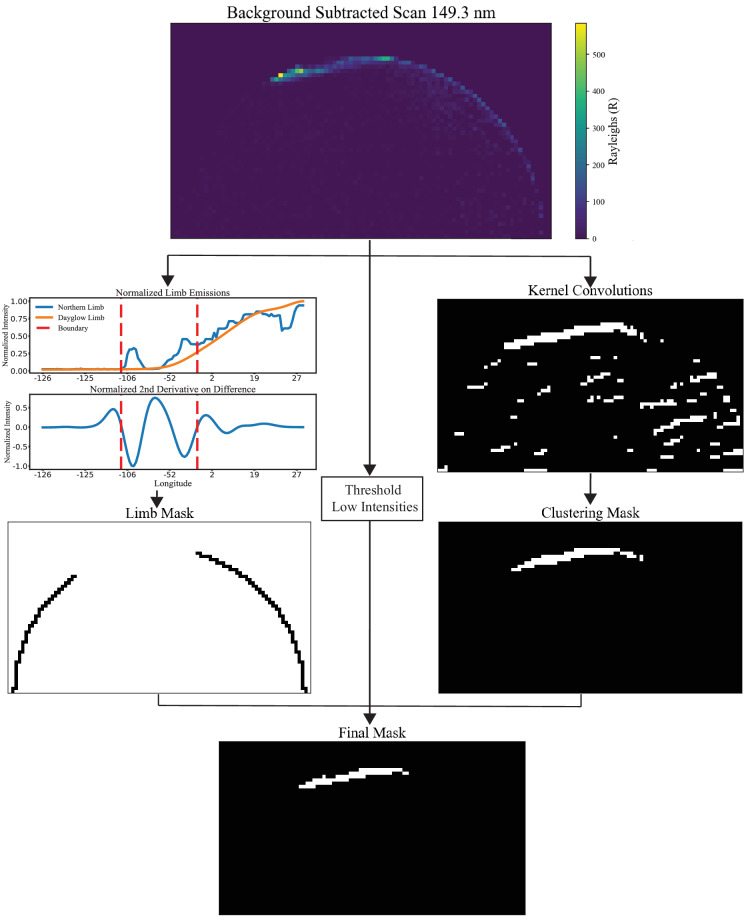
The flow for generating a mask in one instance begins with the differenced image. From this, two routines are applied: a spatial routine (right column) identifies the auroral region, while the 1D signal-based routine (left column) separates the auroral signal from limb emissions. Combining the outputs of both columns, and thresholding for low intensities of the background subtracted image, provides the final mask. The temporal smoothing step of the limb mask is not shown here but is required to ensure accuracy for all mask predictions throughout the day.

A small degree of non-auroral emissions potentially remain in most images after subtraction (e.g. Figure 2 Background Subtraction panel) – necessitating further processing. We developed a three-part classical algorithm that is deterministic in nature (Fig. 3):

#### Image-kernel convolutions

Background-subtracted images are convolved with a Laplacian-of-Gaussian (LoG) kernel to highlight regions of high intensity while suppressing noise^21^. The LoG kernel is a convolution of a Laplacian operator, which measures curvature via the second derivative, with a Gaussian filter that smooths the image. The Laplacian emphasizes relative auroral intensity differences from the background, while the Gaussian mitigates noise.

To further sequester the auroral pixels, the response image from the previous convolution is now convolved with a Gabor filter. Gabor filters are effective at highlighting circular or elongated edge structures, with the response depending on the filter’s orientation. These features are useful since the auroral pixels as seen from GOLD typically are squished near the edge of the disk. To account for temporal variations in auroral morphology, particularly on the nightside, the filter is applied in three discrete orientations that roughly align with morning, noon, and afternoon. The orientations used were selected heuristically based on GOLD local time, rather than derived from an optimization procedure.

In practice the LoG kernel acts as a sharpening filter, and the Gabor filter acts as an edge detector. In the resulting feature maps, responses above the median of the nonzero pixels are set to one, and everything else is set to zero to produce per channel candidate masks.

#### Pixel Clustering

Pixels from the candidate masks are grouped channel-wise using DBSCAN^22^, a density-based clustering algorithm that identifies clusters by requiring a minimum number of neighboring points (min_samples = 10) within a specified distance (eps = 3). We use row & column coordinates of the masks as the input features for clustering. This clustering separates coherent auroral structures from isolated limb pixels or other possible noisy responses from the convolutions. This algorithm may still identify multiple clusters (e.g., limb pixel clusters). To retain only those likely associated with auroral emissions, we keep clusters with a median geographic latitude greater than 50° and classify these as auroral pixels. During extreme geomagnetic events, the auroral oval may extend below this threshold; however, the majority of auroral pixels still remain above it.

#### Auroral–limb boundary refinement

Limb pixels pose the greatest remaining challenge as some of the auroral pixels lie on the limb due to the viewing geometry of GOLD. To disambiguate limb radiances in the northern scans, a predefined limb-pixel mask is applied to the northern scan and the southern scan and each is subsequently flattened to obtain the 1D intensity profile of the limb radiances. The difference between these two signals is typically two gaussian-like peaks in the center, we classify these pixels in between these two peaks as aurora, and everything outside these peaks as limb intensities.

Boundaries are determined by the zero-crossings of the 1D LoG convolved with the differenced signal. The temporal variation of the boundary positions (boundary trajectories) can be determined by combining multiple boundaries vs time. These boundary trajectories are convolved with a 1D median filter to obtain a smooth estimate, under the assumption that auroral boundaries do not vary significantly between consecutive northern scans – separated by approximately 30 minutes.

There is a high degree of variance from the boundary of one instance to the next, however the smooth-estimate trajectory is relatively stable. The assumption for smooth trajectories is used for years with high cadence measurements, 30 minutes between subsequent northern scans. For years after 2021, reduced cadence (~2 h), the previous assumption breaks down and the boundary smoothing is no longer viable for these years. This is a motivation for the Deep Learning-based Segmentation section described below.

Finally, masks from the spatial context (I. & II.), the limb separation (III.), and thresholding low intensities of the background subtracted image are combined channel-wise. To reduce channel-specific noise, a consensus filter retains only pixels identified as auroral in at least two of the three channels, creating a final 2D mask of the aurora. This final mask assumes that the three species have auroral emissions in the same exact pixel location, which is reasonable given the lower resolution of the instrument near the polar regions.

### Deep learning-based segmentation

Accurate boundary smoothing, as described in Section 3, is only possible during years with higher-cadence measurements (2018–2021). After 2021, this assumption breaks down and the previously mentioned mask generation process becomes unstable. To overcome this limitation, and generate masks for later years of the mission, we train a UNet model to directly produce the binary auroral mask predictions.

Here, raw northern scans and classical binary masks outputs from 2020 serve as our image-label training dataset (Fig. 1, step 3). The architecture and training hyperparameters mirror the dayglow model training but with several modifications: the addition of a final layer sigmoid activation to convert to probabilities, binary cross-entropy loss is the objective function, and dropout layers are now included in the architecture for regularization. To better generalize the model, affine transformations (shearing, scaling, rotations, and translations) were applied to the dataset during training, and inputs were standardized per-image, per-channel.

The trained model achieved a Matthews Correlation Coefficient (MCC)^23^ score of 0.893 relative to the classical/intermediate labels on the validation set. The MCC ranges from −1 to 1, where 1 indicates perfect agreement between model and labels, −1 indicates perfect disagreement, and 0 corresponds to no skill. This metric is particularly valuable for imbalanced datasets, such as auroral detection where most pixels are non-auroral, because it accounts for all entries in the confusion matrix.

## Data Records

Data underlying this manuscript are made accessible under a CC0 license through the Virginia Tech Data Repository^24^. The dataset contains over 47,000 unique measurements spanning October 2018 to June 2025.

The dataset is organized by calendar year. Each year’s folder contains individual day files in netCDF (.nc) format. Each day file corresponds to all northern hemispheric scans in a day and contains arrays with the following information:

- Raw GOLD scans in three FUV emission bands: OI 135.6 nm, NI 149.3 nm, and N₂ LBH
- Dayglow estimates representing non-auroral background emissions
- Binary masks identifying auroral pixel locations
- Geographic latitude and longitude calculated at three altitudes: 130 km, 150 km, & 190 km
- Magnetic latitude and magnetic local time (apex quasi-dipole) at 150 km
- Universal Time (UT)
- Solar Zenith Angle (SZA) and Emission Angle (EMA)

Dayside auroral emissions can be obtained by subtracting the provided dayglow estimates from the raw northern scans and applying the binary masks to the background-subtracted data.

The observations derived from the L1C files are mapped onto latitude–longitude grids with a reference altitude of 150 km^25^. This altitude corresponds to the approximate peak emission height for the observed auroral species. However, auroral emissions can span a broader vertical range, which may introduce uncertainties in the inferred geographic and magnetic coordinates^12^. To allow calculations of this uncertainty, we provide additional geographic latitude and longitude grids with reference altitudes of 130 km and 190 km for each pixel. These grids can be used as an estimate of the location uncertainties associated with nominal 150 km altitude assumption.

## Technical Validation

Direct validation of the produced GOLD auroral masks requires comparison with an independent dataset of auroral occurrence. Since there is no concurrent dayside aurora dataset that provides extended overlap in coverage with GOLD, validation is performed against a third party implemented version of the Zhang-Paxton (ZP) auroral oval model^26,27^. The ZP model was derived from nearly four years of FUV auroral images collected by the TIMED/GUVI instrument from 2002–2005, making it a suitable model to compare against GOLD, which also observes in the FUV. GUVI’s orbit enabled sampling of all local time sectors within 60 days, which provided a range of conditions over the 4 years, upon which the model is based. The ZP model is parameterized by Kp and specifies precipitating electron energy flux as a function of magnetic latitude (MLAT) and magnetic local time (MLT) in the Altitude-Adjusted Corrected Geomagnetic Coordinates (AACGM) system^28^. The ZP model poleward and equatorward boundaries are defined by the lowest MLAT of 0.25 ergs cm^−2^ s^−1^ (2.5 × 10^−8^ W cm^−2^) flux, with all enclosed grid cells between these contours classified as aurora. Here we adopt this 0.25 ergs cm^−2^ s^−1^ flux boundary as our auroral threshold for comparison with GOLD.

**Fig. 4:**
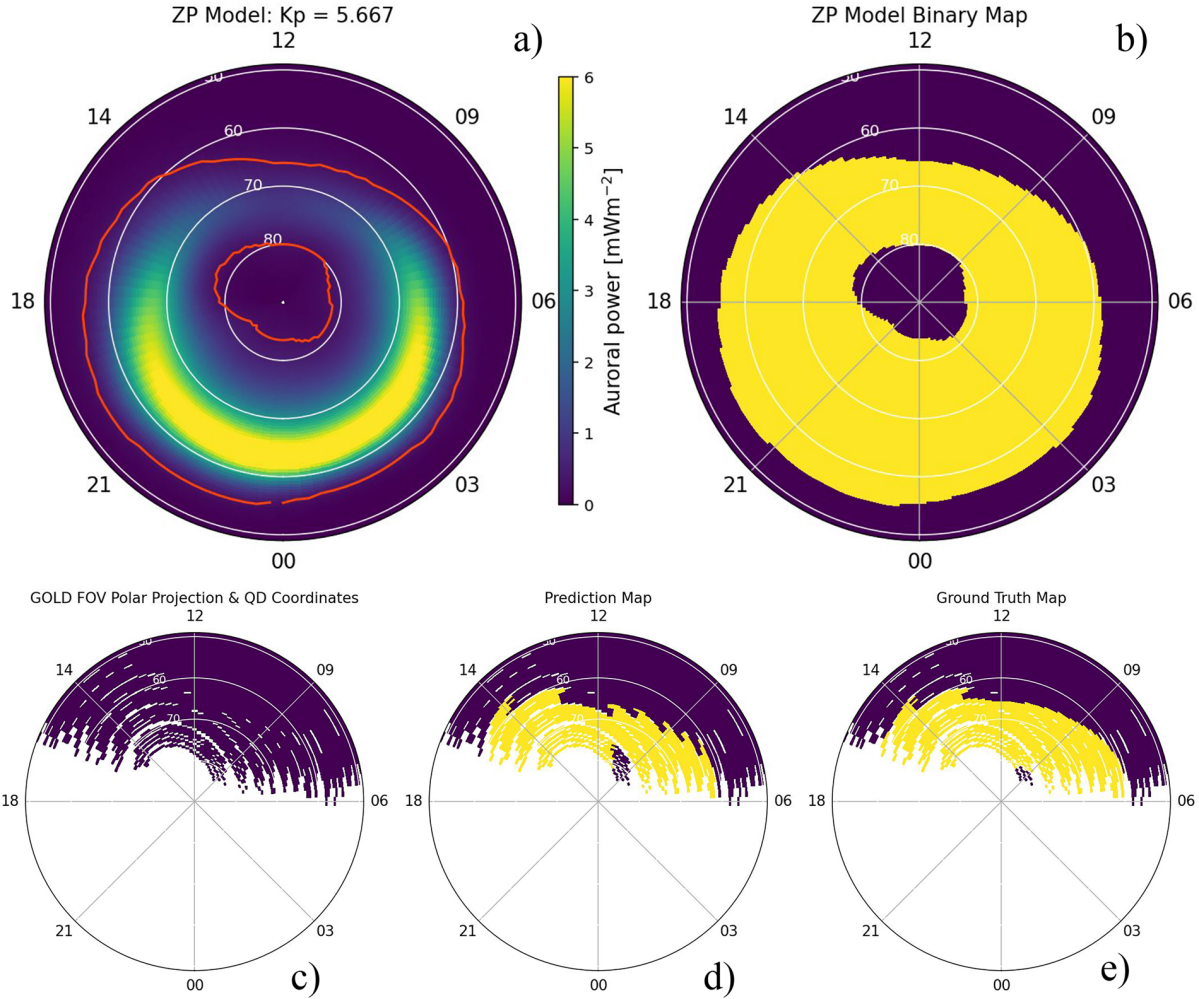
(**a**) Example output prediction of the ZP model at moderate geomagnetic activity. (**b**) Map where all points of the model are above the 0.25 erg cm^−2^ s^−1^ flux threshold. (**c**) Single instance of GOLD’s viewing perspective in quasi-dipole coordinates. (**d**) GOLD binary mask predictions projected onto (**c**) coordinates. (**e**) Ground truth map obtained by combining panels (**c**) and (**d**).

GOLD observations were transformed from geographic to apex quasi-dipole coordinates using the apexpy library^29,30^, with the date of observation as the epoch and at an altitude of 150 km – corresponding to the approximate altitude of the FUV aurora. Although a different geomagnetic coordinate system is used than in the ZP model, the differences between the coordinate systems are negligible at the high magnetic latitudes, where auroral occurrences are concentrated^31^. Therefore, GOLD auroral observations were directly compared to the ZP model auroral classifications at each MLAT/MLT grid point. Since the resolution of GOLD becomes very coarse near the limb, a single GOLD pixel in this region may span a range of auroral and sub-auroral locations. To address this, we compute a finer grid of points within each GOLD pixel — a 5 × 5 array of subpixels — with each subpixel converted to quasi-dipole coordinates. A GOLD pixel is classified as containing an expected auroral signature if at least 3 of the 25 subpixels exceed the 0.25 erg cm^−2^ s^−1^ flux threshold according to the ZP model. An example of this comparison is shown in Fig. 4.

Comparing outputs for a high cadence year (2020), the classical and deep learning methods for segmentation achieved MCC scores of 0.82 and 0.86, respectively, when evaluated against the ZP. The classical method provided reliable identifications of auroral pixels, while the deep learning model, aided by regularization and data augmentation techniques, demonstrated a somewhat stronger agreement with the ZP model. Given this improved performance and its ability to generalize across different geomagnetic conditions, the deep learning model was adopted to generate auroral masks for the complete dataset spanning October 2018 to June 2025.

## Data Availability

The dataset underlying this manuscript is made publicly accessible under a CC0 license through the Virginia Tech Data Repository 10.7294/30179767.v2.

## Code availability

All custom code developed for processing GOLD observations, generating auroral masks, and training models is publicly available with the dataset download or at the following GitHub: https://github.com/jah-26603/dayside_aurora_gold. Model weights are stored with the dataset.
